# Current state of frailty in revision arthroplasty

**DOI:** 10.1186/s43019-024-00245-2

**Published:** 2024-11-27

**Authors:** Brendan Kelly, Nicholas Stratigakis, Arsalaan Sayyed, Tyler K. Williamson, Cameron Atkison, Taylor Manes, Nithin Gupta, Morgan Turnow, Frank A. Buttacavoli

**Affiliations:** 1 Des Moines University College of Osteopathic Medicine, Des Moines, IA USA; 2 State University of New York (SUNY) Downstate Health Sciences University College of Medicine, Brooklyn, NY USA; 3 Department of Orthopaedic Surgery, University of Texas Health San Antonio, San Antonio, TX USA; 4 Department of Orthopedic Surgery, OhioHealth Doctors Hospital, Columbus, OH USA; 5 Campbell University School of Osteopathic Medicine, Lillington, NC USA

## Introductions

The rate of primary total hip arthroplasty (THA) and total knee arthroplasty (TKA) performed has grown substantially in the past few decades and continues to trend upwards within the USA [1]. Across surgical specialties, THA and TKA are among the top five most frequently performed procedures [1]. The reason for this growth is multifaceted, including improved access to care through the growing number of joint surgeons, enhancement of outcomes, and expansion of surgical indications [2, 3]. Given the rise in primary procedures along with the younger patient population, the national rate of aseptic revision has nearly paralleled that of primary joint replacements [4]. In 2014, 50,220 revision total hip arthroplasties (rTHAs) and 72,100 revision total knee arthroplasties (rTKAs) were performed, and Schwartz et al. projected a 43–70% growth in incidence among revision total knee arthroplasty (rTKA) between the years 2014–2030 and a 78–182% growth in rTKA incidence across the same time [5]. It is generally understood, when compared with primary arthroplasty, revision arthroplasty requires longer operative time due to bone loss, scar formation, and unique anatomic considerations, making adequate surgical exposure and implant placement challenging to achieve [6]. Due to these technical challenges, in addition to the aging population, patients undergoing revision arthroplasty are prone to greater morbidity [7]. When compared with primary hip and knee arthroplasty, rTKA and revision total hip arthroplasty (rTHA) have reported a greater risk of sepsis, surgical site infection, and deep surgical site infection, leading to further revisions and risk [8]. These complications cast a heavy burden on the healthcare system as periprosthetic hip and knee infections have been estimated to cost hospitals a combined $1.85 billion by the year 2030 [9]. With the increasing amount of revision procedures performed, risk stratification prior to surgery is of the utmost importance. One methodology that is evolving in orthopedic surgery is using frailty to predict postoperative outcomes [10].

Frailty has been defined as a decline in physiologic reserve and function across multiple domains leading to a lessened capacity to withstand stressors [11]. Patients classified as frail have been associated with heightened vulnerability to complications following orthopedic procedures [11, 12]. As a way of quantifying frailty there has been an emergence of frailty indices in recent literature. Frailty indices have been developed under the accumulation of deficits model consisting of an assortment of factors as a proxy for ageing and mortality [12]. A major benefit to frailty indices is the modifiable nature of its components such as physical activity and nutritional supplementation [13]. This allows for preoperative optimization of patients prior to these elective procedures [14]. Given the myriad of variables contributing to a patient’s frailty, there has been an inconsistency as to which comorbidities should be considered for risk stratification. Multiple frailty indices have been utilized in orthopedic surgery to concisely and effectively quantify patient frailty. For example, the modifiable frailty index (MFI), congestive heart failure, albumin, renal failure on dialysis, dependence for daily living, elderly, and body mass index (CARDE-B), Hospital Frailty Risk Score (HFRS), Age Adjusted Modified Frailty Index (aamFI), and five-factor Modified Frailty Index (mFI-5) are all proven predictors of poor outcomes after revision arthroplasty (Table 1). The main objective of this study is to better understand the current frailty indices and highlight how each instrument has predicted postoperative outcomes in patients undergoing revision arthroplasty.

**Table 1:** Characteristics of included studies

Journal	Source	Study design	*N*	Age cut-off (years)	Surgery types	Frailty measures	Database
International orthopedics	Meyer et al. [15]	Retrospective cohort study	565	No minimum 68.7 ± 12.8 (rTHA) 68.6 ± 9.6 (rTKA) (mean and standard deviation)	rTHA/rTKA	HFRS	Retrospective chart review
The journal of arthroplasty	Zamanzadeh et al. [20]	Retrospective cohort study	32,069	≥ 18 years 66 (median—mean not reported)	rTHA/rTKA	aamFI, mFI-5	NSQIP
The journal of arthroplasty	Kyaw et al. [16]	Retrospective cohort study	47,347	≥ 18 years	rTHA/rTKA	HFRS	NRD
The Journal of Arthroplasty	Tram et al. [17]	Retrospective cohort study	36,243	≥ 18 years	rTHA/rTKA	HFRS CARDE-B	NRD
The journal of bone and joint surgery	Raad et al. [18]	Retrospective cohort study	13,118	No minimum	rTHA/rTKA	HFRS CARDE-B	ACS-NSQIP
The journal of arthroplasty	Traven et al. [19]	Retrospective cohort study	30,252		rTHA/rTKA	mFI-5	Retrospective chart review
Arthroplasty	Momtaz et al. [21]	Retrospective cohort study	17,868	No minimum	rTHA/rTKA	MFI	NSQIP

## Methods

### Search and data sources

A comprehensive literature search was performed using databases PubMed, Scopus, EmBase, and Cochrane Review with the search string of: (frailty) AND (“revision” AND (“arthroplasty” OR “knee” OR “hip” OR “joint”)). Two investigators (N.S. and A.S.) independently used the Rayyan system to first perform a title and abstract screen, followed by a full-text screen to identify studies that met eligibility criteria. Conflicts were resolved by a third independent investigator (BK).

### Selection criteria

The following inclusion criteria were used: (i) related to revision hip and or knee arthroplasty procedures; (ii) use of frailty index to measure patient frailty; (iii) analyzed the effect of frailty on outcomes related to revision arthroplasty procedures in orthopedic surgery; (iv) full text peer reviewed; and (v) published in English language.

The following exclusion criteria were used: (i) not related to revision hip and or knee arthroplasty procedures in orthopedic surgery; (ii) systematic review or literature review; (iii) spinal procedure related; (iv) studies using measure of comorbidity index as the only measure of frailty; (v) does not analyze the effect of frailty on outcomes related to revision arthroplasty procedures; (vi) not full text peer reviewed; and (vii) not published in the English language.

### Data extraction

The following parameters were examined form each study by two blinded investigators: frailty measure, sample size, age cut-off, database, frailty predictive outcomes, factors significantly associated with frailty on multivariable regression, area under the curve (AUC) and comparisons of frailty measure predictive ability to other measures.

### Quality and risk of bias assessment

The Newcastle–Ottawa Quality Assessment Scale (NOS) and scoring assessment was used to assess the methodological quality among all studies included in the systematic review by two reviewers (Table 2).

**Table 2:** Newcastle–Ottawa scale qualitative analysis

		Selection				Comparability	Outcome			
Cohort/case series										
Author and year	Study type	Representativeness of the exposed cohort	Selection of the non-exposed cohort	Ascertainment of exposure	Demonstration that outcome of interest was not present at start of study	Comparability of cohorts on the basis of the design or analysis	Assessment of outcome	Was follow-up long enough for outcomes to occur	Adequacy of follow up of cohorts	Total
Meyer [15]	RC	1	1	1	0	0	1	1	1	6
Zamanzadeh [20]	RC	1	1	1	1	1	1	1	1	8
Kyaw [16]	RC	1	1	1	0	0	1	1	1	6
Tram [17]	RC	1	1	1	0	0	1	1	1	6
Raad [18]	RC	1	1	1	1	0	1	1	1	7
Traven [19]	RC	1	1	1	1	0	1	1	1	7
Momtaz [21]	RC	1	1	1	1	0	1	1	1	7

## Results

### Study selection

**Fig. 1:**
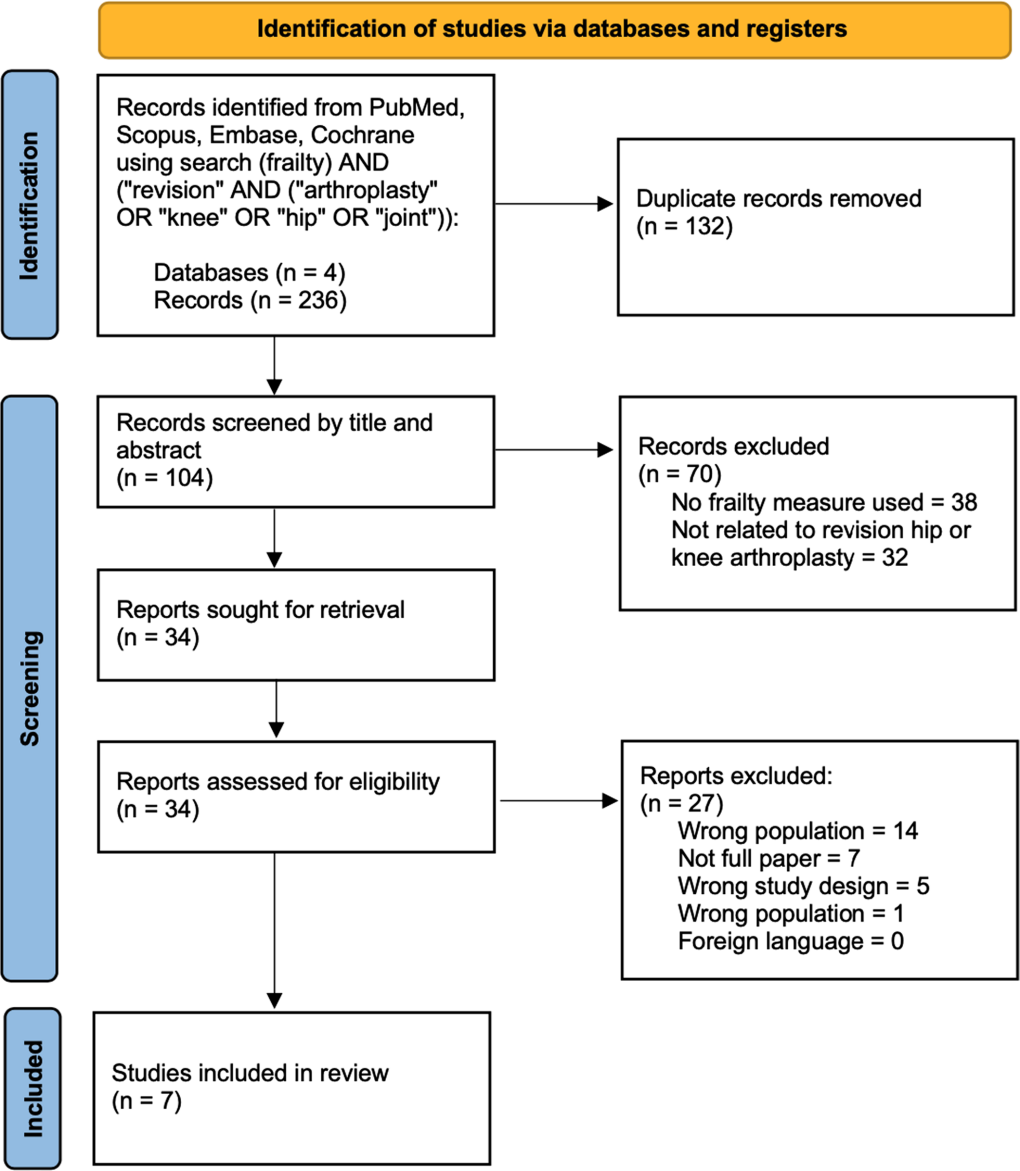
PRISMA flow diagram depicting study selection

The database search identified 236 articles, including 132 duplicate articles, leaving 104 articles to be screened by title and abstract (Fig. 1). From this, 38 articles were excluded due to absence of frailty measure in study and 32 were excluded since they did not focus on revision hip or knee arthroplasties. A total of 34 articles were considered as potentially eligible and were selected for full-text review. Of these, 27 were excluded because they did not meet certain aspects of the inclusion or exclusion criteria as detailed in Fig. 1. A total of seven articles met the criteria for inclusion and were analyzed in the present systematic review.

### Characteristics of included studies

The seven articles that were identified used various frailty measures in a collective total of 177,462 patients who underwent rTKA or rTHA procedures. Three of the studies utilized the HFRS frailty measure only. One of the studies utilized and compared the CARDE-B frailty index to the mFI-5 and American Society of Anesthesia (ASA) measures. One utilized solely the mFI-5 frailty measure. One of the studies utilized and compared the mFI-5 and the aamFI frailty indices. Lastly, one of the studies utilized a modified MFI index (Table 1, 3).

**Table 3:** Characteristics of studies applying frailty indices

Study	Frailty index	Validation cohort	Outcomes						Risk of bias (NOS)
Meyer et al. [15]	HFRS	Retrospective chart review	Surgical	1.156 (0.605–2.210)					6/9 (high)
			Medical	1.851 (0.441–7.769)					
			Overall	1.677 (0.660–4.263)					
Zamanzadeh et al. [20]	aamFI, mFI-5	NSQIP	Any complication	rTHA				2.08 (1.84–2.36)	8/9 (high)
				rTKA				2.36 (2.00–2.78)	
			30-day mortality	rTHA				13.50 (3.18–57.26)	
				rTKA				14.58 (1.92–110.66)	
			30-day unplanned readmission	rTHA				1.19 (0.99–1.43)	
				rTKA				2.02 (1.64–2.51)	
			Non-home discharge	rTHA				5.18 (4.60–5.83)	
				rTKA				3.15 (2.79–3.56)	
Kyaw et al.,[16]	HFRS	NRD	30-day Readmission	Mechanical Loosening			1.97 (1.66–2.32)		6/9 (High)
				Postoperative infection			1.70 (1.50–1.93)		
				Instability			2.09 (1.62–2.66)		
			Prolonged length of stay (LOS)	Mechanical Loosening			1.45 (1.42–1.49)		
				Postoperative Infection			1.71 (1.67–1.75)		
				Instability			1.72 (1.65–1.79)		
			Cost of Hospitalization	Mechanical Loosening			1.14 (1.12–1.16)		
				Postoperative Infection			1.15 (1.13–1.18)		
				Instability			1.20 (1.16–1.24)		
Tram et al. [17]	HFRS CARDE-B	NRD	30-day readmission	Mechanical Loosening			1.88 (1.56–2.25)		6/9 (High)
				Postoperative infection			1.53 (1.36–1.73)		
				Dislocation			1.96 (1.76–2.18)		
			Prolonged LOS	Mechanical Loosening			1.77 (1.72–1.83)		
				Postoperative infection			1.72 (1.68–1.77)		
				Dislocation			1.85 (1.81–1.90)		
			Cost of hospitalization	Mechanical Loosening			1.30 (1.26–1.33)		
				Postoperative infection			1.30 (1.28–1.33)		
				Dislocation			1.32 (1.29–1.34)		
Raad et al. [18]	HFRS CARDE-B	ACS-NSQIP	30-day mortality risk factors		Age > 65 years			6.83 (3.00–15.55)	7/9 (High)
					BMI < 25 kg/m^2^			2.36 (1.46–3.81)	
					Hypoalbuminemia			3.99 (2.38–6.70)	
					Congestive heart failure			4.88 (2.28–10.44)	
					Dependent for activities of daily living			2.67 (1.56–4.57)	
Traven et al. [19]	mFI-5	Retrospective chart review	Adverse discharge		rTKA			1.31 (1.24–1.38)	7/9 (High)
					rTHA			1.29 (1.21–1.36)	
			30-day readmission		rTKA			1.13 (1.03–1.24)	
					rTHA			1.19 (1.09–1.30)	
			30-day mortality		rTKA			1.85 (1.33–2.57)	
					rTHA			1.78 (1.40–2.25)	
Momtaz et al., [21]	MFI	NSQIP	Readmission			2.50 (2.10–3.00)			7/9 (high)
			Complication			3.20 (2.80–3.60)			
			Adverse discharge			3.80 (3.40–4.30)			
			Delayed stay (> 10 days)			5.10 (4.20–6.10)			
			Mortality			18.80 (6.70–52.80)			

### Frailty scales

#### Hospital frailty risk score (HFRS)

The HFRS is a comorbidity measure based on the number of relevant ICD-10 codes from an individual's hospital records [Table 4]. In one retrospective cohort study, researchers grouped patients into three frailty risk categories: low (HFRS < 5), intermediate (5 ≤ HFRS ≤ 15), or high (HFRS > 15) to test whether HFRS is a significant predictor of complication rates. A multivariable logistic regression analysis was performed and revealed that HFRS was independently associated with surgical, medical, and other complications [15].

**Table 4:** Frailty indices used

Hospital frailty risk score (HFRS)	Modified frailty index-5 (mFI-5)	Age-adjusted modified frailty index (aamFI)	CARDE-B	Modified frailty index (MFI)
1. weighted ICD-10 codes	1. Functional status	1. Functional status	1. Congestive heart failure	1. Functional status
	2. History of diabetes	2. History of diabetes	2. Albumin or malnutrition (< 3.5 mg/dL)	2. History of diabetes
	3. Chronic obstructive pulmonary disease	3. Chronic obstructive pulmonary disease	3. Renal failure on dialysis	3. Chronic obstructive pulmonary disease
	4. Congestive heart failure	4. Congestive heart failure	4. Dependence for daily living	4. Congestive heart failure
	5. Hypertension	5. Hypertension	5. Elderly (> 65 years of age)	5. Hypertension
		6. Age (≥ 73 years of age)	6. Body mass index < 25 kg/m^2^	6. Body mass index > 35
				7. Hypoalbuminemia
				8. Osteoporosis

Another study grouped patients into two categories: intermediate/high frailty (5 ≤ HFRS) or low frailty (5 > HFRS). Their multivariate logistic regression analyses showed that high frailty patients had higher odds of 30-day readmission, longer LOS, and greater hospitalization cost. They additionally found that patients with high frailty had significantly higher rates of 30 day reoperation (1.8% versus 1.2%, *p* = 0.01), surgical complications (1.8% versus 1.3%, *p* = 0.02), medical complications (5.0% versus 1.8%, *p* < 0.01), and other complications (1.5% versus 0.6%, *p* < 0.01) [16].

The third study that evaluated the HFRS’s predictive ability among similarly grouped patients into two categories: intermediate/high frailty (5 ≤ HFRS) or low frailty (5 > HFRS). Their multivariate logistic regression analyses similarly showed that high frailty patients had higher likelihood of 30-day readmission, longer lengths of stay, and greater hospitalization costs. Patients falling into the high frailty group had significantly higher rates of 30-day reoperation (9.0% versus 6.1%, *p* < 0.01), surgical complications (11.3% versus 6.9%, *p* < 0.01), medical complications (10.6 versus 3.8%, *p* < 0.01), and other complications (2.0 versus 0.8%, *p* < 0.01) [17].

### CARDE-B frailty index

The CARDE-B Frailty Index (CARDE-B) is a frailty assessment index that is calculated by assigning one point for criteria that predict death in revision total joint arthroplasty (Table 4). Raad et al. analyzed how each factor as well as the cumulative frailty number predicted 30-day mortality following rTHA/rTKA (Table 3). They ultimately found that the AUC for the CARDE-B score in predicting mortality was 0.75. Importantly, they found that the CARDE-B index’s ability to predict 30 day mortality after revision total joint arthroplasty (rTJA) was statistically superior (Table 3) to the ASA physical status classification (AUC: 0.77) and the mFI-5 (AUC: 0.67) [18].

### Modified frailty index (MFI)

The five-item Modified Frailty Index (mFI-5) is a five-factor index that is calculated by assigning one point for each of the included criteria (Table 4). Traven et al. evaluated the ability of the mFI-5 to predict postoperative outcomes in rTHA and rTKA. With regards to rTHA, they found each additional point in the mFI scale increased the risk of a serious medical complication by 147%, whereas the risk for readmission increased by 13.3% per point. Additionally they found the probability of discharge to a facility increased by 31.1% per mFI-5 point, and the risk for mortality increased by 85.1% per point [19].

### Age-adjusted modified frailty index (aamFI)

The aamFI incorporates an additional point into the mFI-5 index: age ≥ 73. Zamanzadeh et al. found that the aamFI is a useful predictor of 30-day complication rate, mortality, readmission and discharge for both rTHA and rTKA. The study found the predictive ability of aamFI for any complication and 30-day mortality after rTKA was an AUC of 0.62 and 0.72, respectively. The ability of aamFI to predict the occurrence of at least one 30-day complication after rTKA was slightly, but statistically superior to mFI-5 (AUC: 0.59; *p* < 0.001). Additionally, the ability of aamFI to predict 30-day mortality after rTKA was statistically superior to mFI-5 (AUC: 0.64; *p* < 0.003) [20]. With regards to rTHA, they also found the ability of aamFI to predict the occurrence of at least one 30-day complication and mortality after rTHA was superior to mFI-5.

Momtaz et al. conducted a similar analysis using a unique modified frailty index (mFI-8) that is calculated by assigning one point or each of the following criteria: non-independent functional status prior to surgery, severe obesity (body mass index > 35), type I or type II diabetes, congestive heart failure within 30 days of surgery, hypoalbuminemia (albumin < 3.5 mg/dL), hypertension requiring medication, history of chronic obstructive pulmonary disease or pneumonia, and osteoporosis. They grouped patients into four categories based on the number of above risk factors: mFI-0 = 0 factors, mFI-1 = 1–2 factors, mFI-2 = 3–4 factors, mFI-3 = 4 or more factors, and compared the outcomes of each group to the original mFI group. They found that, when compared with the original mFI group, the readmission rate, complication rate, discharge, length of stay, and mortality rate were more accurately predicted by the eight-item mFI [21].

### Revision total knee arthroplasty subanalysis

#### Readmission

Meyer et al. examined the effects of intermediate and high HFRS frailty scores compared to low frailty and found rTKA patients with an elevated HFRS to have four times greater odds of 30-day readmission [15]. An aamFI score greater than three was found to have almost three times greater odds of readmission compared to a score of 0 [20]. Traven et al. highlighted patients demonstrated a 13% increase in complication risk with each additional point in the mFI-5 [19].

### Hospital length of stay and discharge disposition

In a study by Zamandazeh et al., patients with an aamFI of 4 were found to have eight times greater risk of non-home discharge after rTKA [20]. Patients with an mFI-5 score of 5 demonstrated double the length of stay compared to a score of 0, and a 1.3 greater odds of non-home discharge with each additional point in mFI-5 [19].

### Overall complications

Patients undergoing rTKA with lower HFRS score demonstrated lower rates of surgical (PJI, periprosthetic fracture), medical (cardiac), and postoperative delirium [15]. Patients with an aamFI of four were found to have four times greater odds of any complication after rTKA [20]. Frail patients undergoing rTKA for infection had two times greater odds of any complication compared to those indicated for loosening or instability [16]. Traven et al. highlighted patients demonstrated a 47% increase in complication risk with each additional point in the mFI-5 [19].

### Mortality

Patients with an aamFI of three or greater were found to have 37 times greater odds of any complication after rTKA, with superior predictability of all-cause mortality compared to the mFI-5 [20]. Traven et al. highlighted patients demonstrated a 85% increase in mortality risk with each additional point in the mFI-5 [19].

## Discussion

Frailty indices grant surgeons the ability to identify which patients are particularly prone to adverse outcomes and, more importantly, which domains require optimization preoperatively. Accuracy in quantifying frailty in the perioperative setting is of value to the orthopedic surgeon as it has proven efficacious in decreasing postoperative complications [22]. The goal of this study was to identify previously described indices when applied to rTHA and rTKA patients.

The common indices utilized for revision arthroplasty are CARDE-B, mFI-5, aamFI, HFRS, and MFI. Each of these indices have proven efficacious in predicting poor outcomes in the revision arthroplasty patient. Stratifying risk of 30-day readmission is of particular interest for the revision arthroplasty patient given the disproportionate cost burden when compared to primary arthroplasty. Bosco et al. found that the mean cost of revision TKA is 1.6 times the cost of a primary TKA, and 2.3 times the cost for revision THA [23]. This outcome has been further compounded by the implementation of Medicare’s Hospital Readmission Reduction Program (HRRP), which withholds up to 3% of reimbursement to hospitals with greater-than-expected 30-day readmission rates for TKA and THA. In our review, the eight-factor mFI by Momtaz et al. demonstrated the highest odds ratio for experiencing 30-day readmission compared to the HFRS and mFI-5 [16, 17, 19, 21]. These findings demonstrate readmission has a significant association with a patient’s baseline frailty. Preoperative assessment and understanding of the potential risk in each of these patients may further prevent these troublesome outcomes for both the patient, hospital systems, and third-party payers.

Indices that consider age, such as the aamFI and CARDE-B, proved particularly useful in predicting 30-day mortality [18, 20]. While frailty, irrespective of age, is a key predictor of mortality, incorporating advanced age has shown to enhance the precision of these tools without complicating their use [20]. Overall, the CARDE-B index demonstrated the greatest discriminative ability for 30 day mortality following rTJA when compared with the mFI-5 and aamFI [18, 20]. This may demonstrate the usefulness in incorporating acute factors into preoperative assessment. The CARDE-B index includes a source to measure nutritional status (albumin) and further validates its association with outcomes following surgery, whereas traditional frailty indices like aaMFI and the mFI-5 are focused solely on chronic conditions [24, 25]

Popularization of the Center for Medicare and Medicaid Services bundled payment models have increased focus on minimizing postoperative complications. In this payment scheme, hospitals and operating surgeons share financial risk for complications within 90 days of surgery [16]. As shown by the results of this review, the eight-item mFI and HFRS performed particularly well in predicting complications within the 30-day postoperative period. Patients who scored high on the HFRS demonstrated the highest odds ratio for experiencing any complication within 30 days of surgery [15]. The optimization of patient care based on frailty has proven beneficial by both improving outcomes and decreasing cost through earlier access to rehabilitation for high-risk patients [26]. Frailty indices such as the HFRS and the eight-item mFI stand to enhance surgeons’ ability to identify at-risk populations and further bolster savings for hospitals, surgeons, and patients.

The increased complexity and worse outcomes of rTJA compared with primary TJA are reflected in revision’s annual cost of $2.7 billion in the USA [27, 28]. Previous studies have attributed over one-third of this cost to non-home discharge [29]. Additionally, in multivariate regression analysis by Owens et al., discharge to a skilled nursing facility (SNF) was an independent risk factor for 30-day complications and readmission to hospital [30]. Similarly, increased LOS in the hospital, as defined by greater than 4 days, has been independently associated with higher 30-day readmission rates [31]. Utilization of frailty indices such as the mFI-5 and the eight-item mFI may assist surgeons in identifying at-risk individuals, allowing for optimization and the reduction in LOS and non-home discharge rates [19, 21]. To our knowledge, other frailty indices have not been validated to predict increased length of stay or non-home discharge for rTJA patients, necessitating further research.

According to our study, mFI-5 and HFRS are the most commonly utilized frailty indices in revision arthroplasty. The modified frailty index and its subdivisions (mFI-5, aamFI) is composed of a series of five to six questions, and its practicality and reproducibility has led many surgeons to utilize it as a modality of risk stratification [20]. The mFI has a strong emphasis on comorbidity status and may better reflect the chronic state of a patient, whereas indices incorporating additional acute variables and functional status may be better linked to certain relevant outcomes, such as disposition and mortality [22]. Conversely, the HFRS also heavily emphasizes comorbidities, but more comprehensively covers other domains of frailty, leading to more significant associations with certain complications. Likewise, HFRS has shown promise for predicting 30-day readmission, LOS, and hospital cost following rTKA and rTHA [15–17]. However, the HFRS is a 109-question survey and, while this provides further granular associations with subcategories of complications, the practicality of this instrument in clinical assessment is severely decreased, especially in the acute setting.

Incorporating a widely accepted and standardized frailty assessment into clinical practice has the potential to greatly improve patient care and outcomes in revision arthroplasty. Given the advantages and limitations of each currently studied index and the absence of a universally recognized gold standard, clinicians should consider selecting frailty indices that best fit their practice environment, diagnoses, and patient population. For instance, concise indices, such as the mFI-5, may be advantageous in settings with limited preoperative resources in the acute setting, while more comprehensive instruments like the HFRS could be better suited for clinic use with access to detailed patient data to properly identify aspects to optimize prior to surgery. By employing an appropriate frailty assessment, clinicians can identify patients at heightened risk and tailor preoperative interventions, including physical therapy (pre-habilitation), nutritional optimization, and targeted management of comorbidities, to mitigate postoperative complications. Given that frailty is a dynamic state, periodic re-evaluation is recommended, particularly for patients awaiting surgery, to capture changes in frailty status and adjust management strategies accordingly. Integrating frailty measures into routine clinical workflows not only enhances surgical risk stratification but also supports the development of individualized patient management plans, thereby underscoring the need for widespread adoption of these tools in clinical practice.

The current study is not without its limitations. The paucity of literature and minimal duplication surrounding frailty indices specific to revision arthroplasty diminishes the generalizability of our findings. In addition to this, most of the studies analyzed (5) utilized national databases which place strict requirements on participating hospitals to ensure quality data. The studies collecting data from their local hospitals via retrospective review may have not been subject to such stringent guidelines, decreasing the quality of data. The national databases, while quality controlled, are limited to 30-day follow up data which is arguably not a sufficient amount of time to gauge post operative success. These pertinent limitations are discussed to highlight the heterogeneity of the current state of frailty in revision arthroplasty. The balance of granularity in survey questions versus heavy reliability on comorbidity status have led to differing limitations for each index. This study stands to highlight the need for a tool that possesses a greater emphasis on all domains of frailty, remains clinically practical, and is capable of predicting a wide variety of complications relevant to the surgery performed.

## Conclusion

Frailty assessment as a means of predicting certain outcomes has proven efficacy when applied to the revision arthroplasty. However, due to the lack of comparative analysis in current literature, each has a unique proven clinical utility without a definitive gold standard for universal assessment. This heterogeneity among frailty scales used for revision total joint arthroplasty has led to inconsistent results and a lack of solidarity, reducing surgeons’ capacity for preoperative optimization and risk stratification.

## Data Availability

N/A.

## Abbreviations

rTHA: Revision total hip arthroplasty
rTKA: Revision total knee arthroplasty
HFRS: Hospital frailty risk score
aamFI: 11-Item modified frailty index
mFI-5: 5-Item ure on dialysis, dependence for daily living, elderly, and body mass index < 25 kg/m^2^
ASA: American society of Anesthesiologists physical status classification system
NSQIP: National surgical quality improvement program
ACS: American college of surgeons
NRD: Nationwide readmission database
CI: Confidence interval
LOS: Length of stay
OR: Odds ratio
BMI: Body mass index
CHF: Congestive heart failure
SC: Surgical complications
MC: Medical complications
OC: Other complications
